# Characteristics of persons with varying vaccination personas: data from the CDC-funded PANDEMIC project

**DOI:** 10.1186/s12982-025-00703-6

**Published:** 2025-06-08

**Authors:** L. B. Cottler, L. C. Rosales, V. L. Seegulam, L. Bilello, C. L. W. Striley, A. Ravenswood, Z. J. Martusewicz, C. E. Murphy, M. Eder, G. Mudd-Martin, L. J. O’Neal, J. Brown Speights, C. Lopez-Quintero, K. D. Everett, A. H. Strelnick, S. Aguilar-Gaxiola, H. Kitzman, J. A. McElroy, T. A. Millay, J. De Leon, L. Warneke, U. K. Weiss, C. O’Leary

**Affiliations:** 1 Department of Epidemiology, College of Public Health and Health Professions and College of Medicine, University of Florida, Gainesville, FL USA; 2 University of Florida Center for Health Equity and Research Engagement, Jacksonville, FL USA; 3 Department of Family Medicine and Community Health, UMN Medical School, University of Minnesota, Minneapolis, MN USA; 4 College of Nursing, University of Kentucky, Lexington, KY USA; 5 Department of Family, Youth and Community Sciences, College of Agricultural and Life Sciences, Institute of Food and Agricultural Sciences, University of Florida, Gainesville, FL USA; 6 Department of Family Medicine and Rural Health, Florida State University, Tallahassee, FL USA; 7 Department of Family and Community Medicine, School of Medicine, University of Missouri, Columbia, MO USA; 8 Department of Family and Social Medicine, Albert Einstein College of Medicine, Bronx, NY USA; 9 Center for Reducing Health Disparities, University of California, Davis, CA USA; 10 Peter O’Donnell Jr. School of Public Health, University of Texas Southwestern Medical Center, Dallas, TX USA; 11 Department of Behavioral Sciences and Social Medicine, Florida State University, Tallahassee, FL USA; 12 Health Literacy Media, St. Louis, MO USA

**Keywords:** Community engagement, Vaccination status, Vaccination personas, Vaccine hesitancy

## Abstract

**Objectives:**

To examine characteristics of personas related to vaccination via a multi-state survey, to better inform strategies to address vaccine hesitancy, foster trust, and promote equitable health outcomes across diverse communities.

**Methods:**

Through a CDC-funded initiative, the University of Florida coordinated and participated with eight partner institutions to increase vaccination uptake and address vaccination hesitancy through trust-building. PANDEMIC (Program to Alleviate National Disparities in Ethnic and Minority Immunizations in the Community) developed an anonymous Survey of Perceptions (SoP), which assessed community perspectives on vaccination. Associations examined persona and gender, state where the interview was conducted, rurality, race/ethnicity, COVID-19 vaccination status, and trusted sources of vaccination information.

**Results:**

From September 18, 2023, to October 3, 2024, over 5,500 participants were surveyed through the SoP to assess general viewpoints on vaccination, with five personas characterized: Enthusiast (58.6%), Watchful (21.0%), Cost-Anxious (10.0%), Skeptic (6.2%), and System Distruster (4.2%). Group comparisons were made using the Kruskal–Wallis rank sum test for continuous variables, and Chi-square tests for categorical variables to assess associations between demographic characteristics and vaccination personas.

**Conclusions:**

Understanding vaccination personas and their predictors may provide a framework for designing targeted public health interventions. By addressing the distinct concerns and motivations of each persona, public health strategies promote equitable health outcomes and increase vaccine uptake across diverse populations.

## Introduction

Vaccination remains one of the most successful public health interventions in preventing infectious diseases. However, vaccine hesitancy—a complex social phenomenon where individuals delay or refuse vaccines despite availability—continues to undermine public health efforts in the United States (US) [1, 2]. Vaccine hesitancy is influenced by a range of cultural, social, psychological, and political factors, which contribute to diverse personal attitudes and behaviors toward vaccines [2–4]. Recent research emphasizes that an individual’s response to getting vaccinated often coalesces into distinct"vaccination personas,"[5–7] which align with unique combinations of beliefs, motivations, and concerns. Identifying the strongest predictors of these personas provides essential insights for tailoring public health strategies to address specific barriers to vaccination. This analysis focuses on five previously described prominent vaccination personas identified among US adults: ‘‘Enthusiasts,’’ ‘‘Watchful,’’ ‘‘Cost-Anxious,’’ ‘‘System Distrusters,’’ and ‘‘Skeptics.’’ These personas may be predicted by varying levels of trust in healthcare, attitudes toward vaccine safety and effectiveness, as well as where someone lives [8–10]. Understanding the underlying predictors of these personas can equip public health practitioners to design interventions that resonate with diverse communities resulting in increased vaccination uptake and reduced health disparities.

Trust in healthcare systems and government institutions plays a significant role in shaping vaccination behaviors across personas. Enthusiasts seek vaccinations as soon as they become available and typically exhibit high trust in medical professionals and government health agencies. The literature indicates that these individuals are often motivated by a strong sense of social responsibility and a desire to protect themselves and their communities [11]. On the other end of the spectrum, System Distrusters rely solely on their judgment rather than healthcare providers or institutions, exemplifying a deep-seated mistrust in the entire medical system. This mistrust often stems from historical and personal experiences with healthcare or governmental organizations, particularly among marginalized groups facing systemic discrimination or inequitable treatment. Research on vaccine hesitancy suggests that personas like System Distrusters are especially resistant to standard health messaging [8, 12].

Social and cultural influences play critical roles in shaping vaccination personas. Vaccination decisions are often influenced by trusted figures within one’s social circle, including family, friends, and community leaders. For instance, the Watchful persona waits and observes others'experiences with vaccinations; people with this persona are frequently influenced by peers and close social circles, suggesting that social modeling and peer influence are key factors in shaping health behaviors within certain cultural and community contexts [13–16]. Cultural icons, religious beliefs, and political ideologies also contribute to vaccine personas, particularly among Skeptics. Research shows that certain religious and political ideologies are associated with skepticism or outright opposition to vaccines, which is synonymous with government overreach [17, 18]. Personas influenced by cultural and political factors are often less responsive to public health messaging from governmental sources, highlighting the need for culturally sensitive communication approaches that respect individual beliefs and/or come from trusted non-governmental sources.

Racial and ethnic minorities, rural residents, and the socioeconomically disadvantaged face barriers to vaccination—extending beyond personal beliefs. Access to healthcare, limited transportation, and lack of paid sick leave are structural barriers disproportionately impacting personas like Cost-Anxious. This persona group is characterized by concerns about vaccine side effects, costs, and the potential for missed work, reflecting the tangible direct and indirect costs of vaccination rather than only intrinsic skepticism. Public health research shows that these practical barriers often reinforce hesitancy, especially among individuals with less financial resources or those residing in medically underserved areas without access to primary and preventive care [19, 20].

Psychological factors, including perceived risk and susceptibility to misinformation, also contribute to individuals who hold beliefs congruent with Skeptic or System Distruster personas. Research highlights that these individuals often have heightened risk perceptions associated with vaccines, shaped by misinformation or conspiracy theories casting doubt on vaccine safety and motives behind vaccination campaigns [1, 4, 21, 22]. Social media platforms have amplified these effects, much like “echo chambers” where skeptical views are reinforced. For Skeptics and Distrusters, traditional health information is often viewed as biased or unreliable, and they are more likely to question the intentions of healthcare providers and government agencies.

By examining the unique characteristics of personas like Enthusiasts, Watchful, Cost-Anxious, Skeptics, and System Distrusters, this analysis provides actionable insights for designing focused public health interventions. While the effectiveness of vaccine interventions across different vaccine personas has not been widely studied, various interventions have shown promise in increasing vaccine uptake in diverse populations, even among those who are hesitant. These interventions include text messaging, nudges, immunization campaign websites, patient-held web-based portals and computerized reminders, and virtual reality interventions, all of which have demonstrated effectiveness in encouraging vaccination uptake [23–25]. Understanding predictors behind each persona type constitutes a first step that can contribute to the development of tailored strategies to address vaccine hesitancy, foster trust, and promote equitable health outcomes across diverse communities.

## Methods

The University of Florida (UF) was one of five sites awarded a CDC initiative to reduce health disparities in adult immunizations. Due to our extensive partnerships with community engagement collaborators, we invited collaborators from the University of California-Davis, Florida State University, University of Kentucky, University of Minnesota, University of Missouri, University of Texas Southwestern Medical Center, and Albert Einstein College of Medicine to partner with us. Additionally, Health Literacy Media (HLM) served as our communications team. Together, we established the PANDEMIC program, which stands for Program to Alleviate National Disparities in Ethnic and Minority Immunizations in the Community. PANDEMIC sought to reach populations of focus defined by the CDC as racial and ethnic minority groups such as Black/African American, Hispanic/Latino(a), Asian American, and Native American. PANDEMIC also expanded outreach to those without health insurance, or who were underinsured, LGBTQ + populations, rural communities, non-English speakers, migrant farm workers, individuals living in or near the poverty line, older adults, individuals who were unhoused, and people with disabilities.

The PANDEMIC project identified 10 Promising Practices, informed by an environmental scan of COVID-19 and other vaccination outreach strategies. Specifically, for the environmental scan, we compiled and analyzed information from published scientific manuscripts and documents that described strategies successfully implemented to reduce vaccine hesitancy and increase vaccine uptake in various settings and diverse populations. This information was discussed and used to refine the 10 Promising Practices, considering trends, potential changes, opportunities, and threats. Our efforts were grounded in the UF HealthStreet model [26], which utilizes Community Health Workers and Promotoras (CHWs/Ps) to engage with community members regularly through outreach events in the community, working alongside trusted neighborhood partners, Cooperative Extension County Educators, and Mobile Health Vehicle (MHV) teams.

One Promising Practice, *Listening to the Community*, involved conducting focus groups and listening sessions with community stakeholders in both English and Spanish and convening a National Community Advisory Board (CAB). Focus groups were conducted with UF IFAS County Extension agents to learn what they were hearing from community stakeholders (e.g., local residents, policy makers, employers, etc.) regarding their concerns about and responses to the COVID-19 pandemic. County agents were recruited from all five Extension districts in Florida and from all Extension programming areas (e.g., agriculture, 4-H youth development, health, etc.) to ensure representation from across the state and from diverse stakeholders. Our National CAB included the co-chair of our UF-FSU Clinical and Translational Science Award CAB as well as a community member representative from each project site. These efforts informed the development of our *Survey of Perceptions* (SoP), which tracked community perspectives on the COVID-19 pandemic. The UF site compiled questions for the SoP that largely came from other assessments used by other national projects; slight modifications were made. These were shared with sites on weekly calls, beta tested and then revised. The SoP was shared on national monthly team calls with other 2113 partners and our CDC Project Officer and minor revisions made. The SoP was pilot tested by all sites at each iteration. All sites used the anonymous SoP, which the CDC considered public health surveillance rather than research, waiving the need for formal informed consent, as determined by the CDC contract and agreed upon by the University of Florida Institutional Review Board. All sites asked participants before beginning if they wanted to participate and because of the anonymous non-research-based protocol, signatures were not allowable. Only persons who verbally agreed to participate were surveyed. Participants completed the survey with the assistance of a CHW/P on paper or through a virtual Qualtrics survey on a mobile computer tablet or QR code by cellphone.

This analysis uses data from the SoP second version, which included data on general vaccination personas (5,538 surveys from 9/18/2023 through 10/3/2024). Data from an earlier version collected information on COVID-19 vaccination personas from 11/15/2021 through 9/17/2023 is not presented here. The SoP also included demographics, trusted sources of vaccination information, and specific COVID-19-related attitudes. Key demographic variables were analyzed, including age, which was captured as a continuous variable (mean age) and categorized into decade-based age groups: young adults (18–29), those in their 30 s, 40 s, or 50 s, and seniors aged 60 and older. Gender was self-reported, including categories for male, female, and transgender/non-binary individuals. Race and ethnicity were reported using six categories: American Indian/Alaska Native/Native Hawaiian or Other Pacific Islander, Asian, African American/Black, White, Bi- or Multi-racial, and Other. The"Other"category captured identities in which the provided options did not fit the participant’s identity, such as Middle Eastern.

Additionally, participants’ states of residence were coded into eight categories (California, Florida, Kentucky, Minnesota, Missouri, New York, Texas, and “Other states”). Nearly 75% (72.5%) of the California surveys included people living in the Sacramento and surrounding area; 89% of the Minnesota surveys came from the Minneapolis/St. Paul, Rochester, and Duluth areas; 51.8% of Missouri surveys came from the Joplin, and Columbia areas with none from the St. Louis metro area; 94.2% of Texas surveys came from the Dallas metro area; 88.7% of the Kentucky surveys included people from Lexington and its surrounding rural areas; and residents from the Bronx made up 80.3% of survey participants. In Florida, 36.5% of the surveys were from Tallahassee and its surrounding rural areas, while 46.4% came from Gainesville and surrounding areas (82.9% of Floridian participants). People living in other states (n = 115) came from across the country (frontier states, rural states, urban areas) with only one of these additional states having more than 20 people (with an average of 5 people in the others). Rurality was classified using ZIP Code Tabulation Areas (ZCTAs) to distinguish between rural and urban respondents. A derived variable, Populations of Focus (PoF), was also created, identifying individuals who were non-White or Hispanic/Latino, aligning with CDC guidelines of prioritizing these groups for recruitment. Respondents were queried on COVID-19 vaccination status (vaccinated or not), plans to get a COVID-19 vaccine in the future (yes, no, or unsure), and self-reported non-COVID vaccination status (categorized as up-to-date, not up-to-date, or unsure).

Participants’ attitudes toward vaccination were categorized into five distinct personas, adapted from the Surgo Ventures persona scale [27]. These included: The Enthusiasts, who get vaccinated as soon as possible; The Watchful, who prefer to wait and observe how others respond to vaccines; The Cost-Anxious, concerned about side effects, such as illness or missing work; The System Distrusters, who lack trust in the healthcare or vaccination system; and The Skeptics, who doubt the need for vaccines in general. Respondents were also asked to indicate their most trusted source of information about vaccinations in an open-ended format; responses included doctors or other healthcare providers, CDC/FDA or other government agencies, co-workers, family members, friends, scientists, and researchers, news, religious leaders, or nobody (only trust myself). This allowed for the examination of trust dynamics in vaccination decision-making processes among our PoF.

### Statistical analyses

All data cleaning and analyses were conducted using R Studio (version 2024.09.1 + 394). Descriptive statistics were generated to summarize the demographic characteristics and vaccination personas of the study population. Continuous variables, such as age, were reported as means with standard deviations, while categorical variables, including gender, race/ethnicity, and vaccination personas, were presented as frequencies and percentages. Group comparisons were made using the Kruskal–Wallis rank sum test for continuous variables, given the non-normal distribution of age, and Chi-square tests for categorical variables to assess associations between demographic characteristics and vaccination personas. Significance was set at p < 0.05.

## Results

Over 5500 participants were surveyed about their vaccination persona with 58.6% of respondents identified as Enthusiasts, 21.0% as Watchful, 10.0% as Cost-Anxious, 6.2% as Skeptics, and 4.2% as System Distrusters (see Table 1). The most frequented outreach event venues included churches, temples, or other faith-based/religious sites, libraries, community recreation centers, schools, colleges, community colleges, trade schools, and parks.

**Table 1:** Sociodemographic characteristics from the Survey of Perceptions by Viewpoints on General Vaccination (September 18, 2023, through October 3, 2024, N = 5,538)

	Enthusiasts	Watchful	Cost Anxious	Skeptics	System Distrusters		p-value^a^
	I got vaccinated as soon as I could	I am a person who waits to do something until I see what happens to others	I worry about the vaccine making me sick and not being able to work or do things I normally do	I am skeptical about the need for vaccines	I do not trust the system	Total	
	n = 3,244 (58.6%)	n = 1,161 (21.0%)	n = 553 (10.0%)	n = 345 (6.2%)	n = 235 (4.2%)	N = 5538	
Mean Age (SD)	48.7 (18.8)	41.9 (17.2)	44.2 (16.8)	49.4 (16.8)	44.8 (15.7)	46.7 (18.3)	< 0.0001
Young Adults (18–29)	705 (21.7%)	356 (30.7%)	132 (23.9%)	47 (13.6%)	46 (19.6%)	1286 (23.2%)	< 0.0001
Thirties (30–39)	446 (13.7%)	224 (19.3%)	117 (21.2%)	68 (19.7%)	51 (21.7%)	906 (16.4%)	
Forties (40–49)	452 (13.9%)	182 (15.7%)	75 (13.6%)	53 (15.4%)	44 (18.7%)	806 (14.6%)	
Fifties (50–59)	503 (15.5%)	174 (15.0%)	105 (19.0%)	59 (17.1%)	50 (21.3%)	891 (16.1%)	
Seniors (60 +)	1138 (35.1%)	225 (19.4%)	124 (22.4%)	118 (34.2%)	44 (18.7%)	1649 (29.8%)	
Gender							
Male	920 (28.4%)	372 (32.0%)	143 (25.9%)	110 (31.9%)	89 (37.9%)	1634 (29.5%)	0.0018
Female	2277 (70.2%)	782 (67.4%)	409 (74.0%)	235 (68.1%)	145 (61.7%)	3848 (69.5%)	
Transgender or Non-binary	47 (1.4%)	7 (0.6%)	1 (0.2%)	0 (0%)	1 (0.4%)	56 (1.0%)	
State where the Respondent Lived							
California	296 (9.1%)	121 (10.4%)	28 (5.1%)	26 (7.5%)	16 (6.8%)	487 (8.8%)	0.0005
Minnesota	769 (23.7%)	208 (17.9%)	43 (7.8%)	32 (9.3%)	21 (8.9%)	1073 (19.4%)	
Missouri	62 (1.9%)	37 (3.2%)	19 (3.4%)	24 (7.0%)	28 (11.9%)	170 (3.1%)	
Texas	623 (19.2%)	194 (16.7%)	58 (10.5%)	59 (17.1%)	29 (12.3%)	963 (17.4%)	
Kentucky	146 (4.5%)	144 (12.4%)	27 (4.9%)	6 (1.7%)	5 (2.1%)	328 (5.9%)	
Florida	946 (29.2%)	371 (32.0%)	351 (63.5%)	168 (48.7%)	110 (46.8%)	1946 (35.1%)	
New York	320 (9.9%)	74 (6.4%)	21 (3.8%)	24 (7.0%)	17 (7.2%)	456 (8.2%)	
Other States	82 (2.5%)	12 (1.0%)	6 (1.1%)	6 (1.7%)	9 (3.8%)	115 (2.1%)	
Rurality							
Urban	2263 (69.8%)	776 (66.8%)	323 (58.4%)	235 (68.1%)	149 (63.4%)	3746 (67.6%)	< 0.0001
Rural	981 (30.2%)	385 (33.2%)	230 (41.6%)	110 (31.9%)	86 (36.6%)	1792 (32.4%)	
Ethnicity							
Non-Hispanic or Latino(a)	2325 (71.7%)	742 (63.9%)	423 (76.5%)	291 (84.3%)	204 (86.8%)	3985 (72.0%)	< 0.0001
Hispanic or Latino(a)	919 (28.3%)	419 (36.1%)	130 (23.5%)	54 (15.7%)	31 (13.2%)	1553 (28.0%)	
Race							
American Indian/Alaska Native/Native Hawaiian or Other Pacific Islander	90 (2.8%)	36 (3.1%)	14 (2.5%)	13 (3.8%)	9 (3.8%)	162 (2.9%)	0.0005
Asian	276 (8.5%)	88 (7.6%)	12 (2.2%)	6 (1.7%)	10 (4.3%)	392 (7.1%)	
AA/Black	873 (26.9%)	321 (27.6%)	286 (51.7%)	130 (37.7%)	80 (34.0%)	1690 (30.5%)	
White	1757 (54.2%)	552 (47.5%)	197 (35.6%)	177 (51.3%)	127 (54.0%)	2810 (50.7%)	
Biracial/Multiracial	57 (1.8%)	19 (1.6%)	17 (3.1%)	7 (2.0%)	3 (1.3%)	103 (1.9%)	
Other	191 (5.9%)	145 (12.5%)	27 (4.9%)	12 (3.5%)	6 (2.6%)	381 (6.9%)	
Population of Focus (Non-white or Hispanic/Latino)							
No	1125 (34.7%)	330 (28.4%)	120 (21.7%)	148 (42.9%)	112 (47.7%)	1835 (33.1%)	< 0.0001
Yes	2119 (65.3%)	831 (71.6%)	433 (78.3%)	197 (57.1%)	123 (52.3%)	3703 (66.9%)	
COVID-19 Vaccination Status							
Not Vaccinated	177 (5.5%)	189 (16.3%)	228 (41.2%)	164 (47.5%)	164 (69.8%)	922 (16.6%)	< 0.0001
Vaccinated	3067 (94.5%)	972 (83.7%)	325 (58.8%)	181 (52.5%)	71 (30.2%)	4616 (83.4%)	
Do you plan to get COVID-19 vaccines in the future?							
No	295 (9.1%)	360 (31.0%)	212 (38.3%)	230 (66.7%)	198 (84.3%)	1295 (23.4%)	< 0.0001
Yes	2463 (75.9%)	407 (35.1%)	177 (32.0%)	34 (9.9%)	16 (6.8%)	3097 (55.9%)	
Not sure	486 (15.0%)	394 (33.9%)	164 (29.7%)	81 (23.5%)	21 (8.9%)	1146 (20.7%)	
Non-COVID vaccination status							
Not Up-to-date	233 (7.2%)	159 (13.7%)	66 (11.9%)	90 (26.1%)	91 (38.7%)	639 (11.5%)	< 0.0001
Up-to-date	2795 (86.2%)	845 (72.8%)	441 (79.7%)	207 (60.0%)	112 (47.7%)	4400 (79.5%)	
Not Sure if Up-to-date	216 (6.7%)	157 (13.5%)	46 (8.3%)	48 (13.9%)	32 (13.6%)	499 (9.0%)	
Who do you trust the most to give you information about vaccinations?							
Doctor or other healthcare provider(s)	1787 (55.1%)	671 (57.8%)	306 (55.3%)	157 (45.5%)	68 (28.9%)	2989 (54.0%)	0.0005
Government Agencies or Researchers	1051 (32.4%)	251 (21.6%)	59 (10.7%)	43 (12.5%)	20 (8.5%)	1424 (25.7%)	
Nobody (only trust myself)	66 (2.0%)	77 (6.6%)	99 (17.9%)	86 (24.9%)	102 (43.4%)	430 (7.8%)	
Family or Peers	236 (7.3%)	116 (10.0%)	69 (12.5%)	34 (9.9%)	23 (9.8%)	478 (8.6%)	
News or Internet	74 (2.3%)	29 (2.5%)	10 (1.8%)	11 (3.2%)	6 (2.6%)	130 (2.3%)	
Religious or Community Leader(s)	30 (0.9%)	17 (1.5%)	10 (1.8%)	14 (4.1%)	16 (6.8%)	87 (1.6%)	

^a^For categorical variables with normal distributions, the Pearson Chi-Square test was performed; for categorical variables with non-normal distributions, the Pearson Chi-Square test using simulated p-values calculated over 2000 replications; for continuous variables, the Kruskal–Wallis rank sum test

Overall, the average age of participants was 46.7 years (SD = 18.3), with seniors representing the largest age group (> 59 years, 28%) and young adults (18–29 years) accounting for 23.2%. The gender distribution showed that the majority of participants were female (69.5%), with males comprising 29.5% and transgender or non-binary individuals representing 1.0%. The two states with the largest number of participants were Florida (35.1%), followed by Minnesota (19.4%). Nearly one-third (32.4%) of the population was from a rural area.

In terms of racial and ethnic diversity, the sample included a significant proportion of minority groups according to CDC guidelines that prioritized recruiting the PoF. Among the respondents, 28.0% identified as Hispanic/Latino. Regarding race, 30.5% identified as Black or African American, and 7.1% as Asian. Whites made up 50.7% of the total sample.

Regarding vaccination status, 83.4% of the population reported having received a COVID-19 vaccination *(“Did you ever receive a COVID-19 vaccine?” yes/no)* and over half (55.9%) of the participants reported that they"plan to get a COVID-19 vaccine in the future."Of note: at the time of survey data collection, up to 8 opportunities had been offered for vaccination: 1 st COVID shots (beginning in December 2020), 1 st booster (~ September 2021), 2nd booster (~ March 2022), bivalent shot (~ September 2022), bivalent booster or single dose (~ April 2023), 2023 annual shot (~ September 2023), 2024 booster (~ March 2024), and 2024 annual shot (~ September 2024). An additional 20.7% said they were unsure if they would get a COVID-19 vaccine in the future. Nearly 80% (79.5%) said they were up to date on their non-COVID vaccines while 9% said they were not sure of their status. When asked “Who do you trust most to give you information about vaccinations?” 54.0% said their doctor or other healthcare provider; 25.7% said a government agency or researcher (mostly the CDC and FDA).

When comparing all personas, the Skeptics were the oldest on average (49.4 years; SD = 16.8). The Watchful tended to be the youngest (41.9 years; SD = 17.2). The Enthusiasts had the highest percentage of seniors, those 60 + years of age (35.1%). The System Distrusters had the least variability in age, among the decades of age, ranging from 15.7% to 21.7%. Females were most represented in the Cost-Anxious group (74.0%), and least represented in the System Distruster group (61.7%). System Distrusters had the highest percentage of males (37.9%).

Comparing state variation in personas, our findings suggested that Californians (5.1%), Texans (10.5%), and New Yorkers (3.8%) were least likely to be represented in the Cost-Anxious group, while Floridians were most likely to be in the Cost-Anxious group (63.5%). Minnesotans were most likely to be in the Enthusiast group (23.7%) and Kentuckians were most likely to be represented in the Watchful group (11.9%) Missourians were most represented among the System Distrusters (11.9%). There was a preponderance of rural participants in the Cost-Anxious group (41.6%) compared to all other personas (30.2% to 36.6%).

When evaluating ethnicity, the Watchful group had the largest proportion of Hispanics/Latinos (36.1%), followed by the Enthusiast group (28.3%). Across all personas, Black/African-Americans (51.7%), Whites, (35.6%, and the biracial group (3.1%) were most represented in the Cost-Anxious group. The “Other” race group was most represented in the Watchful persona (12.5%). The Cost-Anxious group had the highest proportion of PoF (78.3%); the System Distrusters group had the lowest proportion of PoF (52.3%). 

**Fig. 1:**
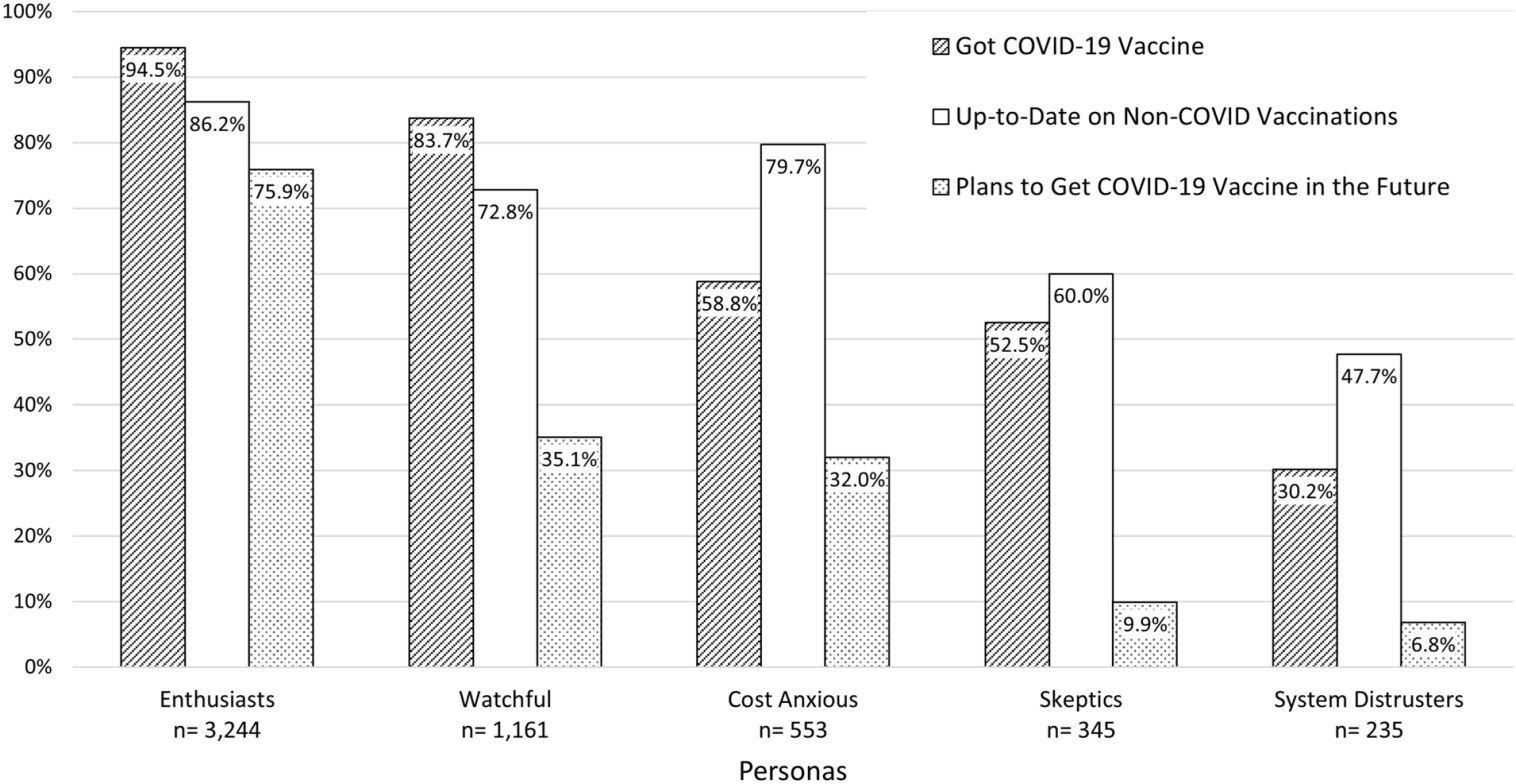
Bar graph depicting the percentage of the three vaccination statements by vaccination personas (9/18/2023 to 10/3/2024, N = 5538)

A distinguishable trend in the association between vaccination persona and COVID-19 vaccination status was observed, with Enthusiasts having the highest rate of reporting having had a COVID-19 vaccination (94.5%) followed by the Watchful (83.7%), the Cost Anxious (58.8%), the Skeptics (52.3%) and the System Distrusters having the lowest (30.2%). The same trend was found for those planning to get a COVID-19 vaccine in the future, with three-fourths of Enthusiasts reporting planning to get a COVID-19 vaccine in the future, versus only 9.9% and 6.8% among Skeptics and System Distrusters, respectively. A similar relationship was found for *non-COVID* vaccination status, with 86.2% of Enthusiasts reporting being up to date on their non-COVID vaccinations and the lowest rate among the System Distrusters at 47.7%. One additional finding to note: while 47.7% of System Distrusters were up to date on non-COVID vaccinations, only 30.2% reported being vaccinated with the COVID-19 vaccine. On the other hand, while 72.8% of the Watchful group was up to date on non-COVID vaccines, 83.7% of this group reported having been vaccinated for COVID-19 (see Fig. 1).

Except for the System Distrusters, participants from four other personas reported trusting their doctor or other healthcare provider most to give them information about vaccinations. System Distrusters mainly reported trusting only themselves for information about vaccinations (43.4%) compared to only 2% among the Enthusiasts. The Cost-Anxious group was most likely versus all other groups to trust their family and peers (12.5%). Finally, Skeptics and System Distrusters were more likely than any other group to report trusting religious and community leaders (4.1% and 6.8%, respectively vs. 9.0% to 1.8% among the other personas).

## Discussion

At a time when vaccine hesitancy remains a prominent topic across the country [28–31], it is increasingly critical to understand the characteristics of individuals who eagerly accept vaccinations as well as those who are skeptical of them or distrust healthcare systems in general. Insights into these differences are essential for developing effective, targeted, interventions to address and reduce hesitancy. From September 18, 2023, until October 3, 2024, we surveyed individuals about their perspectives on general vaccinations to understand how best to increase uptake for COVID-19 and other vaccines. This analysis characterized five prominent vaccination personas—Enthusiasts, Watchful, Cost-Anxious, System Distrusters, and Skeptics—each shaped by distinct predictors. Characterizing these personas offers a novel view of the varying levels of trust, concerns, and barriers associated with vaccination decisions and provides actionable insights for designing tailored public health interventions for the future.

Both Skeptics (6.2% of the sample) and System Distrusters (4.2%) require unique approaches to increase vaccine confidence. Skeptics generally question the need for vaccines due to underlying beliefs, often shaped by cultural, social, or political factors. These individuals may be more susceptible to misinformation, particularly within social media"echo chambers"that reinforce doubt. Addressing Skeptics requires interventions that not only counteract misinformation with clear, factual messaging but also respectfully address their concerns to avoid entrenching their skepticism further. Our data suggest that connecting with faith and community leaders may be one way to intervene.

System Distrusters, likely to be male from the Midwest, are not up to date on their COVID-19 or non-COVID-19 vaccinations. People with this persona exhibit deeper trust issues, particularly with healthcare institutions and government agencies, often trusting no one but themselves. Traditional health messaging may be ineffective for this group. Instead**,** community-based approaches that engage trusted local figures—such as community health workers or religious leaders—may be more impactful, as these individuals hold credibility within a System Distrusters’ community [32, 33]. In fact, people with this persona did report trusting religious leaders most. Studies indicate that bypassing institutional authority in favor of community-centered engagement is an effective approach [34–36].

Health literacy is another factor impacting vaccination attitudes. Studies show that individuals with lower health literacy are more likely to fall into hesitant personas, as they struggle to access, understand, and trust information about vaccine safety and effectiveness [32, 37]. There was a 40.8 percentage point difference between the Enthusiasts and the Watchful group (highest- 75.9% and next highest- 35.1%) who plan to get a COVID-19 vaccine in the future. Unlike the Enthusiasts and the Watchful, the Cost-Anxious, Skeptic, and System Distruster personas reported higher rates of being up to date on non-COVID-19 vaccinations than for COVID-19 vaccinations.

Effective interventions to increase vaccine confidence must account for the unique characteristics of each vaccination persona. Enthusiasts and Watchful individuals respond positively to messaging that emphasizes community benefits and social responsibility, while Cost-Anxious individuals benefit from strategies that address logistical concerns, such as providing paid leave or mobile vaccination clinics in underserved areas. Literature suggests that counteracting misinformation with fact-based messaging can help mitigate risk perceptions among those with a hesitant persona, though this approach must be carefully designed to avoid further distrust [1, 4, 21, 22]. In addition, research has shown that repeated exposure to misinformation or disinformation can influence the perceived accuracy of the statement(s), known as the illusory truth effect [38–42]. Therefore, crafting responses to misinformation must also attend to message design to counteract the illusory truth effect. For instance, while vaccine-associated reactions do occur, some of the more serious reactions receive more media attention which tends to normalize the message and minimize the more credible messages [43].

A major strength of this study is its comprehensive, multi-site sample allowing for detailed analysis across diverse demographic groups and geographic regions. This diverse sampling approach enhanced the study’s generalizability and relevance to underrepresented populations identified by the CDC. Additionally, the use of CHWs/Ps in survey administration increased engagement, particularly among marginalized groups, which likely enhanced the quality and trustworthiness of the data collected [44–47].

Although doctors and other healthcare providers were the most trusted source of information for four of the five personas, the COVID-19 response did not prioritize vaccinations with trusted primary care physicians, but rather prioritized speed and convenience by distributing the COVID-19 vaccines through chain pharmacies and large volume public health- and hospital-based vaccination and testing centers. Federally qualified community health centers sought, successfully, to provide both trusted providers and access to vaccinations for the low-income population of focus.

The PANDEMIC study had several limitations. First, the cross-sectional design limited our ability to infer causality; thus, while we identified associations between personas and vaccination behaviors, we could not establish temporal relationships. Longitudinal studies could provide deeper insights into how these personas evolved over time or in response to external factors, such as public health messaging, community experiences with health outcomes from infection, or changes in vaccine access. Second, while we strived to achieve a representative sample, the reliance on self-reported data introduced potential biases, including social desirability or recall bias, which may have influenced respondents'reporting of vaccination attitudes and behaviors. Additionally, while this study considered important social determinants and trust dynamics, other unmeasured factors—such as specific religious or ideological beliefs and individual health literacy—may have also played a significant role in shaping personas. Language barriers among non-English speakers further compounded this issue, making it essential to design communication strategies that were clear, accessible, culturally relevant, and available in multiple languages to accommodate diverse populations. While we conducted surveys in English and Spanish only, incorporating other languages could have refined the characterization of each persona and improved the precision of tailored interventions.

Despite these limitations, our findings highlight the importance of targeted, persona-based approaches to public health interventions aimed at increasing vaccine confidence and uptake. Moving forward, integrating longitudinal designs and expanding the scope of demographic and attitudinal variables will be valuable for enhancing the robustness of these personas. Future research should also explore interventions that address systemic barriers among System Distrusters and practical barriers for Cost-Anxious individuals, such as accessible vaccine locations and paid sick leave policies. Addressing these factors will help ensure that public health strategies are responsive to the complex, evolving landscape of vaccine attitudes, as well as ongoing difficulties with preventive care access, ultimately supporting efforts to reduce health disparities and improve vaccine uptake across diverse communities [48].

In conclusion, understanding vaccination personas and their predictors provides a valuable framework for designing targeted public health interventions. By addressing the distinct concerns and motivations of each persona, public health strategies can promote equitable health outcomes and increase vaccine uptake across diverse populations.

## Data Availability

The data that support the findings of this study will be available from the University of Florida's Centers for Disease Control and Prevention (CDC) funded PANDEMIC grant in December 2025 through a Data Use Agreement by contacting the first author.
